# Silencing of microRNA-135b inhibits invasion, migration, and stemness of CD24^+^CD44^+^ pancreatic cancer stem cells through JADE-1-dependent AKT/mTOR pathway

**DOI:** 10.1186/s12935-020-01210-1

**Published:** 2020-04-25

**Authors:** Jingyang Zhou, Haihong Wang, Jinhui Che, Lu Xu, Weizhong Yang, Yunjiu Li, Wuyuan Zhou

**Affiliations:** 1 Class 182, Queen Mary School, Medical Department, Nanchang University, Nanchang, 330031 People’s Republic of China; 2 Department of Hepatopancreatobillary Surgery, Xuzhou City Cancer Hospital, No. 131 Huancheng Rd., Gulou District, Xuzhou, 221000 Jiangsu People’s Republic of China

**Keywords:** microRNA-135b, Jade family PHD finger 1, Protein kinase B/mammalian target of rapamycin pathway, Pancreatic cancer, Stemness

## Abstract

**Background:**

Recent studies have emphasized determining the ability of microRNAs (miRNAs) as crucial regulators in the occurrence and development of pancreatic cancer (PC), which continues to be one of the deadliest malignancies with few effective therapies. The study aimed to investigate the functional role of miR-135b and its associated mechanism to unravel the biological characteristics of tumor growth in pancreatic cancer stem cells (PCSCs).

**Methods:**

Microarray analyses were initially performed to identify the PC-related miRNAs and genes. The expression of miR-135b and PCSC markers in PC tissues and cells was determined by RT-qPCR and western blot analysis, respectively. The potential gene (JADE-1) that could bind to miR-135b was confirmed by the dual-luciferase reporter assay. To investigate the tumorigenicity, migration, invasion, and stemness of PC cells, several gain-of-function and loss-of-function genetic experiments were conducted. Finally, tumor formation in nude mice was conducted to confirm the results in vivo.

**Results:**

miR-135b was highly-expressed in PC tissues and PCSCs, which was identified to specifically target JADE-1. The overexpression of miR-135b promoted proliferation, migration, and invasion of PCSC, inhibited cell apoptosis and increased the expression of stemness-related factors (Sox-2, Oct-4, Nanog, Aldh1, and Slug). Moreover, miR-135b could promote the expression of phosphorylated AKT and phosphorylated mTOR in the AKT/mTOR pathway. Additionally, miR-135b overexpression accelerated tumor growth in nude mice.

**Conclusions:**

Taken together, the silencing of miR-135b promotes the JADE-1 expression, which inactivates the AKT/mTOR pathway and ultimately results in inhibition of self-renewal and tumor growth of PCSCs. Hence, this study contributes to understanding the role of miR-135 in PCSCs and its underlying molecular mechanisms to aid in the development of effective PC therapeutics.

## Background

Pancreatic cancer (PC) is a devastating malignancy afflicting the digestive system [1] with a 5-year survival rate of less than 10% over the past decades [2]. According to GLOBOCAN 2018, PC remains one of the most lethal malignancies leading to 432,242 new deaths in 2018, as patients rarely present symptoms before the advanced stage of the disease [3, 4]. Currently, it was reported that there is a subpopulation of cells in tumors, cancer stem cells (CSCs), which are capable of self-renewal and generating unique progeny that form the main tumor cell component of tumors [5]. Thus, the possible reason for the high incidence of PC can be associated with the high oncogenic ability of pancreatic CSCs (PCSCs) which can not only boast self-renewal ability but also produce later generation with ability to differentiate [6]. Intriguingly, the functional role of PSCSs in promoting the metastasis of PC could be related to the resistance of PC to standard chemotherapy and radiation treatment [7]. There is a well-defined requirement to investigate more potential therapeutic approaches for the treatment of PC.

Located on 1q32.1, miR-135b is encoded in the first intron of the LEMD1 gene, cancer/testis antigen [8]. Specifically, miR-135b is a well-documented oncogene, possesses the ability to adjust the growth of various tumors [9]. Moreover, several studies have illustrated the aberrant expression of miR-135b in various malignant tumors, which highlights the crucial role of miR-135b in multiple tumors [10, 11]. Of note, a Global microRNA expression profiling of microdissected tissues has indicated the potential role of miR-135b might act as a novel biomarker for pancreatic ductal adenocarcinoma [12]. Additionally, another study by Jiang et al. has confirmed the involvement of miR-135b in tumourigenesis and chemoresistance of PC [13].

Nevertheless, due to its high expression in renal proximal tubules, JADE-1 has been suggested as a promising candidate for renal tumor inhibitor [14] that accelerates the apoptosis of renal cancer [15]. Importantly, the AKT/mammalian target of rapamycin (mTOR) is known as one of the most critical pathways in regulating cell development, proliferation, and survival [16]. A further study has indicated that the activation of the AKT/mTOR pathway could enhance cell proliferation in PC cells [17]. Besides, another study suggested that the AKT/mTOR pathway could also impact the survival ability and proliferation of prostate cancer cells [18]. Additionally, the AKT pathway could be regulated by miR-135b in colorectal cancer cells [19]. However, scarce data is determining the relationship between miR-135b and JADE-1 and the effect of this correlation on PC progression. Considering these limitations here we aimed to investigate whether miR-135b target JADE-1 to regulate the AKT/mTOR pathway, thus modulating the invasion, migration, and stemness of PCSCs.

## Materials and methods

### Ethics statement

All the animal study was carried out by following the Institutional Animal Ethics Care and Use Committee of Xuzhou City Cancer Hospital. Extensive efforts were made to minimize the pain and suffering of the experimental animal.

### Bioinformatics prediction of PC related miRNA/gene

PC-related datasets were obtained from the Gene Expression Omnibus (GEO) database (https://www.ncbi.nlm.nih.gov/geo/) for differential expression analysis. One miRNA expression dataset (GSE41369) and three coding gene expression datasets (GSE16515, GSE32676, and GSE71989) were used to screen out differentially expressed miRNAs/genes. Details of the datasets are shown in Additional file 1: Table S1. The R-language affy installation package (http://www.bioconductor.org/packages/release/bioc/html/affy.html) was applied to carry out standardized pre-processing of datasets. The limma package (http://master.bioconductor.org/packages/release/bioc/html/limma.html) was used to screen out differentially expressed miRNAs/genes between tumor tissues and normal tissues and the corrected *p*-value was expressed by adj. *p*. Val. The miRNAs/genes with |log2FC| > 1.0 and adj. *p*. Val < 0.05 were considered significantly differentially expressed whereas the heat map of differentially expressed genes was drawn with heatmap package (https://cran.r-project.org/web/packages/pheatmap/index.html). The target genes of differentially expressed miRNAs were predicted in the DIANA, miRDB, miRWalk, and mirDIP. Then, miRNA-gene pairs differentially expressed in PC and regulatory interactions were screened in connection with the Venn online analysis tools-Calculate and custom Venn diagrams were drawn (http://bioinformatics.psb.ugent.be/webtools/Venn/).

### Cell culture

Human PCSCs (CD44^+^/CD24^+^/ESA^+^) were isolated from human PC cell line PANC-1 (cell bank of Chinese Academy of Sciences, Shanghai, China) (source bank: https://www.fenghbio.cn) according to the method described by Jun Sheng Fu et al. [20]. The isolated PCSCs were cultured in the PCSC culture medium (Celprogen) containing 1% N2, 2% B27, 100 ng/mL epidermal growth factor, and 1% antifungal agent (Invitrogen, Shanghai, China), as well as 20 ng/mL human platelet, derived growth factor (Sigma-Aldrich, Shanghai, China). The cells were cultured in a 37°C incubator with 5% CO_2_ and 95% saturated humidity [21].

### Cell grouping and transfection

CD24^+^CD44^+^ESA^+^ cells were assigned into the agomir-negative control (NC) group, the agomir-miR-135b group, the antagomir-NC group, the antagomir-miR-135b group, the sh-NC group, the sh-JADE-1 group, the dimethyl sulphoxide (DMSO) group (10 μmol/mL, St. Louis, MO, USA), the perifosine (AKT/mTOR inhibitor) group (10 μmol/mL, Selleck Chemicals, CA, USA), the agomir-miR-135b + DMSO (10 μmol/mL) group, and the agomir-miR-135b + perifosine (10 μmol/mL) group. The antagomirs and agomirs were designed and synthesized by Dharmacon Inc. (Lafayette, CO, USA).

CD24^+^CD44^+^ESA^+^ PCSCs were seeded into a 6-well plate and incubated overnight. The cells were then treated with EntransterTM-R transfection solution comprised of agomir-miR-135b, antagomir-miR-135b, agomir-NC, and antagomir-NC.

Lentiviral vector pLKO.1 (SCH002) was purchased from Sigma-Aldrich (Shanghai, China). The short hairpin (sh)-JADE-1 and its corresponding control vector were constructed by Wuhan Miaolingbio Inc. (Hubei, China) to establish a stable cell line. The polybrene (6 mg/mL, Sigma-Aldrich, Shanghai, China) was added to the transfected cells with the aforementioned lentivirus. After 72 h of transduction, the cells were screened with 2 mg/mL puromycin for 4 days.

### Dual-luciferase report gene assay

The 3′ untranslated region (3′UTR) fragment of JADE-1 was amplified by PCR and cloned into the pmirGLO vector (Promega, Madison, WI, USA) by Shanghai Sangon Biotech Co., Ltd. (Shanghai, China) to construct wild-type (WT) and mutant-type (MUT) recombinant dual-luciferase reporter plasmids. The 293T cells (American Type Culture Collection [ATCC]) were assigned into 4 groups and co-transfected with miR-135b or NC and pmirGLO-JADE-1-WT 3′UTR or pmirGLO-JADE-1-MUT 3′UTR, respectively. After 30 h, the 293T cells were collected, followed by determining the luciferase activity using a fluorescent light detector (Promega Glomax 20/20, Yuanpinghao Biotechnology Co. Ltd., Beijing, China) according to the provided instructions of the dual-luciferase reporter gene detection kit (Promega, Madison, WI, USA).

### Reverse transcription-quantitative polymerase chain reaction (RT-qPCR)

Total RNA was extracted based on the Trizol method (Invitrogen, Carlsbad, California, USA). Total RNA was then reversely transcribed into cDNA using the RevertAIDTM First Strand cDNA Synthesis Kit (Fermentas, Thermo Fisher, NY, USA). The expression of miR-135b was measured using a TaqMan miRNA assay (Ambion, Austin, TX, USA) with U6 regarded as an internal reference. The expression of JADE-1 was determined by the PrimeScript RT-PCR kits (TaKaRa, Shiga, Japan) and glyceraldehyde 3-phosphate dehydrogenase (GAPDH) was employed as an internal reference. All primers were synthesized by Shanghai GeneCore BioTechnologies Co., Ltd. (Shanghai, China) as illustrated in Table 1. The fold changes were calculated using relative quantification (2^-ΔΔCt^ method).

**Table 1:** RT-qPCR primer sequences

miRNA/gene	Primer sequence
miR-135b	F: 5′-CTGTGGCCTATGGCTTTTCAT-3′
	R: 5′-GCTCGCCCCTCACTGTAG-3′
U6	F: 5′-CGCTTCGGCAGCACATATAC-3′
	R: 5′-AAATATGGAACGCTTCACGA-3′
JADE-1	F: 5′- AGGGATTAAAGTGCTCCCCC-3′
	R: 5′-TGGCGTTTGGCTTGTTATGG-3′
GAPDH	F: 5′-AATGGGCAGCCGTTAGGAAA-3′
	R: 5′-GCGCCCAATACGACCAAATC-3′
CD24	F: 5′-CCCCACCTTGCCTGCG-3′
	R: 5′-AAATCTGCGTGGGTAGGAGC-3′
CD44	F: 5′-TTACAGCCTCAGCAGAGCAC-3′
	R: 5′-TGACCTAAGACGGAGGGAGG-3′
ESA	F: 5′-TGGTTTCAGGGGGCTGTTGT-3′
	R: 5′-TCGGCCGTCTCTACGTCCTC-3′
Sox-2	F: 5′-GCCTGGGCGCCGAGTGGA-3′
	R: 5′-GGGCGAGCCGTTCATGTAGGTCTG-3′
Oct-4	F: 5′-GCTCGAGAAGGATGTGGTC-3′
	R: 5′-ATCCTCTCGTTGTGCATAGTCG-3′
Nanog	F: 5′-GCTGAGATGCCTCACACGGAG-3′
	R: 5′-TCTGTTTCTTGACTGGGACCTTGTC-3′
ALDH1	F: 5′-GCACGCCAGACTTACCTGTC-3′
	R: 5′-CCTCCTCAGTTGCAGGATTAAAG-3′
Slug	F: 5′-CGAACTGGACACACATACAGTG-3′
	R: 5′-CTGAGGATCTCTGGTTGTGGT-3′

*RT-qPCR* reverse transcription quantitative polymerase chain reaction, *microRNA-135b* miR-135b, *JADE-1* jade family PHD finger 1, *GAPDH* glyceraldehyde 3-phosphate dehydrogenase, *ESA* electrical stimulation for analgesia, *F* forward primer, *R* reverse primer, *Sox-2* SRY related HMG box-2, *Oct-4* Octamer-4, *ALDH1* aldehyde dehydrogenase isoform 1

### Western blot analysis

The total protein in cells was extracted by Radio Immunoprecipitation Assay lysis buffer containing phenylmethane sulfonyl fluoride (R0010, Beijing Solarbio Science & Technology Co., Ltd., Beijing, China). The protein concentration was determined according to the instructions of the bicinchoninic acid kit (20201ES76, Yeasen Company, Shanghai, China). Each well was loaded with 30 μg sample and the protein was separated by the polyacrylamide gel electrophoresis and transferred into a polyvinylidene fluoride membrane using a wet transferring method. The membrane was blocked for 1 h with 5% bovine serum albumin at room temperature. The primary antibodies to anti-human phosphorylated AKT (1:1000; ab38449; Abcam Inc., Cambridge, UK), anti-human AKT (1:1000; ab38449; Abcam Inc.), anti-mTOR (1:5000; ab137133; Abcam Inc.), anti-phosphorylated mTOR (1:1000; ab109268; Abcam Inc.), anti-human cleaved-caspase-3 (1:1000; Cell Signaling Technology, Beverly, MA, USA), anti-human total caspase-3 (1:1000; #9662; Cell Signaling Technology), anti-human cleaved-caspase-9 antibody (1:1000; 20,750; Cell Signaling Technology), anti-human total caspase-9 (1:1000; 9502; Cell Signaling Technology), anti-SRY related HMG box-2 (Sox-2; 1:1000; 2748; Cell Signaling Technology), anti-octamer-binding transcription factor 4 (Oct-4; 1:1000; 2750; Cell Signaling Technology), Nanog rabbit monoclonal antibody (1:5000; ab109250; Abcam Inc.), acetaldehyde dehydrogenases (Aldh1) rabbit monoclonal antibody (1:1000; ab52492; Abcam Inc.), anti-Slug antibody (5 µg/mL; ab51772; Abcam Inc.), JADE-1 rabbit polyclonal antibody (1:1000; ab155215; Abcam Inc.) and GAPDH rabbit polyclonal antibody (1:2500; ab9485; Abcam Inc.) were cultured with the membrane overnight at 4 °C, followed by 1 h of incubation with horseradish peroxidase-labeled corresponding secondary antibody at room temperature. By applying the electrochemiluminescence (ECL) fluorescence detection kit (No. BB-3501, AmerSham GE Healthcare, Shanghai, China), the membrane was placed into a gel imager for exposure imaging. The membranes were then photographed using the Bio-Rad image analysis system (Bio-Rad, Inc., Hercules, CA, USA) and subsequently analyzed using the Quantity One v4.6.2 software. The relative protein content was expressed by the ratio of the gray value of the target protein band to that of the GAPDH protein band.

### Cell counting kit-8 (CCK-8)

The CCK-8 proliferation kit (Dojindo, Kumamoto, Japan) was used for cell proliferation detection. The cells were treated with 0.25% trypsin, followed by the addition of Dulbecco’s modified eagle’s medium (DMEM) to the triturate into individual cells. Following after, the cells were resuspended in stem cell medium for subsequent use. After the cell density was adjusted, the cells were seeded into a 96-well plate with 1000 cells per well in 100 μL culture medium, and the plate was gently rotated to achieve uniform dispersion. The cells were then cultured in an incubator under stable conditions of 37 °C with 5% CO_2_ with saturated humidity. A total of 10 μL CCK-8 reagent was added at 0 h, 12 h, 24 h, 48 h and 72 h, respectively, shaken gently, and mixed evenly. After incubation at 37 °C for 2 h, the optical density (OD) of each well was measured using an enzyme-linked immunosorbent assay (450 nm measuring wave). Each group experiment was performed in quintuplicate with a minimum of three independent experiments.

### Soft agar colony formation assay

Cells in the logarithmic growth phase in each group were detached using 0.25% trypsin and triturated into individual cells. The cells were then suspended in DMEM with 10% fetal bovine serum (FBS), seeded into a 6-well plate, and subsequently cultured at 37 °C with 5% CO_2_ with saturated humidity. The culture medium was changed at regular intervals of 4 days. The cells were then fixed with a 4% polyformaldehyde solution after 2 weeks, stained with 0.1% crystalized violet (Sigma-Aldrich, Shanghai, China), and counted manually under a microscope. The experiment was repeated 3 times.

### Transwell assay

Cell migration and invasion assessments were conducted using the Transwell chamber (Boyden Chambers; Corning, Cambridge, MA, USA). The cells were suspended in serum-free medium (5 × 10^5^ cells/mL). Cell migration assay was performed as follows: 200 µL cell suspension was added to the apical chamber of Transwell. In the cell invasion experiment, 200 µL cells were suspended in the apical chamber of Transwell covered with Matrigel matrix glue (Corning, Cambridge, MA, USA). All 600 µL of DMEM containing 10% FBS was added to the basolateral chamber of the transwell. After the cells were incubated for 24–48 h, the cells in the apical chamber were erased using cotton swabs, fixed with 4% polyformaldehyde solution, and stained with crystalized violet for 10 min (Sigma-Aldrich, Shanghai, China). An inverted microscope (CX22; Olympus, Tokyo, Japan) was employed to randomly select 5 fields to observe, photograph, and subsequently count the cell number. The experiment was repeated 3 times [22].

### Flow cytometry

Flow cytometry was used to detect cell apoptosis. The cells were seeded in a 6-well plate, and cultured for 48 h after treatment of virus plasmids. The cells were treated with trypsin, washed twice with phosphate-buffered saline (PBS), and then resuspended in PBS (200 μL) containing 10 μL RNAase (10 mg/mL) for incubation at 37 °C for 30 min. Afterward, a 50 μL propidium iodide (PI) solution was added and stored in an environment void of light at room temperature for 20 min. The whole experiment was conducted on the flow cytometer (Accuri C6, BD Biosciences, San Jose, CA, USA) [23]. The upper right quadrant represented late apoptotic cells, and the lower right quadrant represents early apoptotic cells. Apoptosis rate = late apoptosis rate + early apoptosis rate.

### Onco-spheroids formation assay

The adherent cells were treated with 0.25% trypsin to prepare a single-cell suspension. The cell density was adjusted to 1 × 10^3^ cells/mL, and then seeded into growth medium containing stem cells (adding 1% N2, 2% B27, 100 ng/mL epidermal derived growth factor, and 1% antifungal agent [Invitrogen, Carlsbad, California, USA]; 20 ng/mL human platelet growth factor, [Sigma-Aldrich, Shanghai, China]) in a low adsorption 6-well plate (Corning Inc., Corning, NY, USA) with 2 mL/well. The cells were then subsequently cultured to obtain suspended cell spheres, and semi-quantitative liquid exchange was performed every 2 days. After such a continuous culture for 10 days, the number of newly formed suspended cell spheres per well were recorded and averaged under a microscope. The experiment was repeated 3 times [20, 21].

### In vivo study

A total of 36 BALB/C male nude mice (aged 4–6 weeks; weighing 18–20 g) were purchased from the experimental animal center of Southern Medical University (Guangdong, China) with all mice confirmed to have passed the quarantine inspection. The nude mice were randomly divided into agomir-NC, agomir-miR-135b, antagomir-NC, antagomir-miR-135b, agomir-miR-135b + DMSO, and agomir-miR-135b + perifosine groups (n = 6 in each group), raised in specific pathogen-free (SPF) animal houses. The successfully constructed and stably-transfected cells (1 × 10^6^ cells) were mixed with 75 μL matrix gel (50:50; Beckon Dickinson, Bedford, MA, USA). All 100 μL prepared tumor cell suspension was injected into the subcutaneous flanks of nude mice by a 1 mL-syringe. The tumor volume (V) = L (length) × W^2^ (width) × 0.5 was observed every 3 days and measured by a vernier caliper. After 30 days, the nude mice were euthanized and the subcutaneous tumors were completely stripped and weighed.

### Immunohistochemistry

The paraffin-embedded sections were dewaxed and hydrated: sections were immersed in xylene I and II for 10 min, respectively, and in a different gradient of alcohol (100%, 95%, 80%, and 70%) for 2 min, respectively. The sections were immersed in 3% H_2_O_2_ for 10 min followed by 10-min antigen retrieval. The sections were then cooled down at room temperature, and supplemented with 5% bovine serum albumin, followed by the 30-min culture at 37 °C. The sections were then cultured with 50 μL rabbit polyclonal antibodies (Abcam, Cambridge, UK) to phosphorylated AKT (1:500, ab38449), JADE-1 (1:500, ab155215), and phosphorylated mTOR (1:100, ab2732) overnight at 4 °C. Then, goat anti-rabbit immunoglobulin G antibody (1:1000, ab6721, Abcam) was supplemented to culture the sections at 37 °C for 30 min. Afterward, the sections were developed by diaminobenzidine and counterstained with hematoxylin followed by dehydration, clearing, sealing, and observation under a microscope. PBS buffer instead of the primary antibody was used as NC. The proportion of positive cells more than 10% was regarded as positive. The staining was mainly located in the cytoplasm or membrane or cytoplasm, which was brownish-yellow. Five high power visual fields were randomly selected to observe the positive expression rate of phosphorylated AKT, JADE-1, and phosphorylated mTOR.

### Statistical analysis

All data were processed using the SPSS 21.0 statistical software (IBM Corp. Armonk, NY, USA). Measurement data were expressed as the mean ± standard deviation. Normal distribution and homogeneity of variances were used to assess all data. A paired student’s *t*-test was used to compare paired data between the two groups. Whereas unpaired student’s *t*-test was used to compare unpaired data between two groups. One-way analysis of variance (ANOVA) was applied for comparison among multiple groups followed by Tukey’s post hoc test. Two-way ANOVA was performed to compare the data of cell viability among multiple groups at different time points. Repeated-measurement ANOVA was used to compare the data among multiple groups at different time points, followed by Bonferroni post hoc test. A *p* < 0.05 value was considered to be indicative of statistical significance.

## Results

### miR-135b is highly expressed in PCSCs

**Fig. 1:**
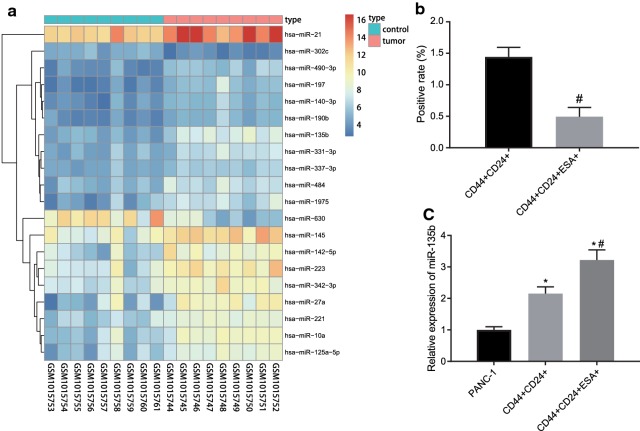
miR-135b was highly expressed in PCSCs. **a** The heat map of top 20 differential miRNA expression in PC miRNA dataset GSE41369 (the abscissa indicated the sample number, the ordinate indicated the differential gene, the histogram at the top right indicated the color scale and each rectangle in the map corresponded to a sample expression value); **b** The proportion of flow sorting positive cells in PANC-1 cells; C, RT-qPCR was used to detect miR-135b expression in cells; *p < 0.05 vs. the PANC-1 group, #*p* < 0.05 vs. the CD44^+^CD24^+^ group. The measurement data were expressed as mean ± standard deviation. Unpaired student’s *t*-test was used to compare unpaired data between two groups. One-way ANOVA was applied for comparison among multiple groups, followed by Tukey’s post hoc test. The experiment was repeated 3 times

Bioinformatics was applied to predict the potential molecular targets of PC. R language was used to screen differentially expressed miRNA/genes from PC datasets. Firstly, the differentially expressed miRNAs were selected from miRNA dataset GSE41369. According to the ascending order of adj. *p*. Val, we ranked the top 20 differentially expressed miRNAs in GSE41369 (Fig. 1a). Among these miRNAs, miR-135b was highly expressed in PC tumor tissues (log FC > 1 and adj. *p*. Val was the smallest, Additional file 1: Table S2). To study the difference of miR-135b expression in PC cells and PCSCs, the flow cytometry was utilized to categorize the three-positive PCSCs and RT-qPCR was conducted to detect the expression of miR-135b in both PC cells and PCSCs. Our results showed that (1.442 ± 0.152)% of the PANC-1 cells were CD44^+^CD24^+^, while only (0.492 ± 0.149)% of the cells were CD44^+^CD24^+^ESA^+^ (Fig. 1b). The expression of miR-135b in CD44^+^CD24^+^ and CD44^+^CD24^+^ESA^+^ cells was significantly higher than that in PANC-1 cells, but the expression of miR-135b in CD44^+^CD24^+^ESA^+^ cells was significantly higher than that in CD44^+^CD24^+^ cells (Fig. 1c, *p* < 0.05). Therefore, these results suggested that miR-135b exhibited high expression in PCSCs.

### miR-135b inhibitor suppresses viability while enhances apoptosis of PCSCs

**Fig. 2:**
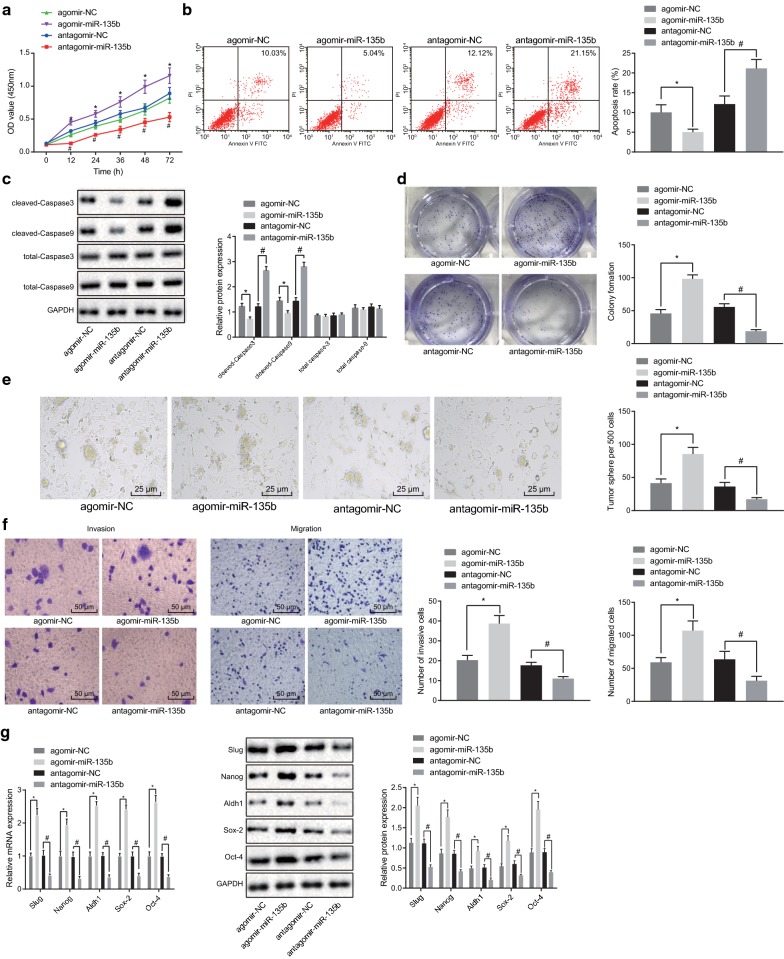
miR-135b inhibition could suppress viability, invasion, and migration and promote apoptosis in PCSCs. **a** CCK-8 assay was used to test the viability of PCSCs. **b** Flow cytometry was applied to detect apoptosis of PCSCs. **c** Western blot analysis was utilized to detect the expression of apoptotic proteins. **d** The cloning ability of PCSCs was tested by soft agar colony forming experiment. **e** onco-spheroids formation assay was used to detect the sphere-forming ability of PCSCs (×400). **f** Transwell assay was applied to examine the migration and invasion of PCSCs (×200). **g** Western blot analysis and RT-qPCR were used to detect expression of tumor stem cell markers. *p < 0.05 vs. the agomir-NC group; ^#^*p* < 0.05 vs. the antagomir-NC group. The measurement data were expressed as the mean ± standard deviation. One-way ANOVA was applied for comparison among multiple groups, followed by Tukey’s post hoc test. Two-way ANOVA was performed to compare the data of cell viability among multiple groups at different time points. The experiment was repeated 3 times

To study the effect of miR-135b on the biological characteristics of PCSCs, the viability of PCSCs was measured using the CCK-8 method. Our results showed that agomir-miR-135b promoted cell viability (Fig. 2a, *p* < 0.05). Meanwhile, the apoptosis of PCSCs was detected by flow cytometry whereas the expression of apoptosis-related proteins (total caspase-3, total caspase-9, cleaved-caspase-3, and cleaved-caspase-9) was determined by western blot analysis. Our results showed that miR-135b significantly inhibited the apoptosis in PCSC and the expressions of cleaved-caspase-3 and cleaved-caspase-9 were decreased accordingly, but no significant difference in expression of total caspase-3 and total caspase-9 after different transfections were found (Fig. 2b, c, *p* < 0.05). Thereafter, the cloning ability of PCSCs was tested and we found that miR-135b elevated the colony formation of cells (Fig. 2d, *p* < 0.05). The above-reported results demonstrated that overexpression of miR-135b could promote the proliferation of PCSCs and inhibit cell apoptosis.

Onco-spheroids formation assay was used to test the sphere-forming ability of PCSCs. Our results exhibited that compared with the agomir-NC group, the sphere-forming ability of the agomir-miR-135b group was significantly enhanced. However, compared to the antagomir-NC group, the ability of stem cells to form spheres in the antagomir-miR-135b group was markedly decreased (Fig. 2e, *p* < 0.05). Transwell assay was employed to examine the migration and invasion of PCSCs and results indicated that the migration and invasion of PCSCs in the agomir-miR-135b group were significantly increased compared with the agomir-NC group. Moreover, compared to the antagomir-NC group, the migration and invasion of stem cells in the antagomir-miR-135b group were significantly reduced (Fig. 2f, *p* < 0.05). Western blot analysis and RT-qPCR analyses were used to detect the mRNA and protein expression of tumor stem cell markers (Sox-2, Oct-4, Nanog, Aldh1 and Slug). Intriguingly, our results indicated that the overexpression of miR-135b could promote the mRNA and protein expression of these tumor stem cell markers (Fig. 2g, *p* < 0.05). Taken together, our results indicated that the downregulated miR-135b could affect the cellular physiology of PCSCs.

### Low expression of miR-135b inhibits tumorigenesis and tumor growth *in vivo*

**Fig. 3:**
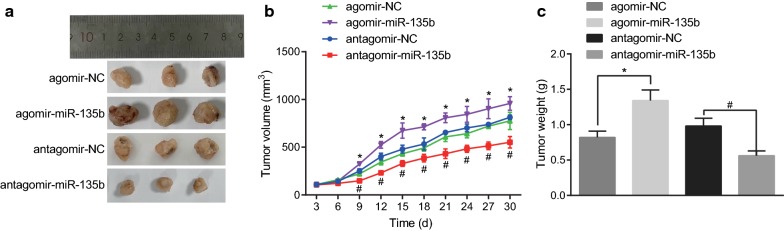
Inhibited miR-135b suppressed tumorigenesis and tumor growth in vivo. **a** Macroscopic observation of tumor size on 30th day. **b** measurement of tumor volume in nude mice with vernier caliper. **c** Determination of tumor mass in nude mice by weighing method; The tumorigenicity and growth of PCSCs were detected by tumor formation in nude mice. n = 6, *p < 0.05 vs. the agomir-NC group, ^#^*p* < 0.05 vs. the antagomir-NC group; The measurement data were expressed as mean ± standard deviation. One-way ANOVA was applied for comparison among multiple groups, followed by Tukey’s post hoc test. Repeated measurement ANOVA was used to compare the data among multiple groups at different time points, followed by the Bonferroni post hoc test

To identify the tumorigenicity and tumor growth of PCSCs with low expression of miR-135b, tumor formation assay in nude mice was performed using PCSCs transfected with the agomir-miR-135b, the agomir-NC, the antagomir-miR-135b, and the antagomir-NC respectively. The growth of tumor in nude mice was analyzed by recording the growth, volume, and weight of tumor-bearing mice subcutaneously. The tumor weight was measured by peeling off the tumor tissue on the 30th day. Our results manifested that compared with the agomir-NC group, the tumor growth rate of PCSCs in nude mice of the agomir-miR-135b group, the PCSCs in nude mice were significantly accelerated, the tumor volume was obviously increased, and the tumor weight was markedly raised. However, compared with the antagomir-NC group, the subcutaneous tumors of nude mice in the antagomir-miR-135b group grew slowly whereas the tumor volume and weight were remarkably reduced (Fig. 3a–c, *p* < 0.05). These results indicated that the low expression of miR-135b in PCSCs inhibited tumorigenesis in vivo.

### miR-135b is predicted to target JADE-1 to regulate PC progression

**Fig. 4:**
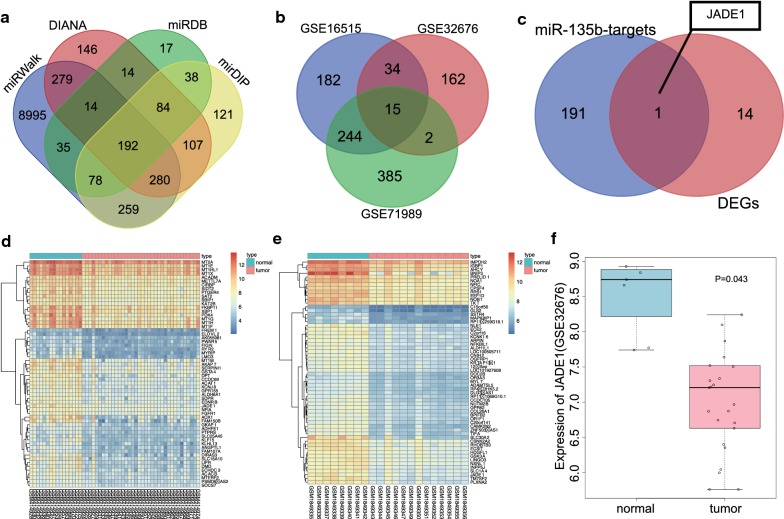
JADE-1 was predicted to be a target gene of miR-135b. **a** Comparison among the results of predicting miR-135b target genes from DIANA, miRDB, miRWalk and mirDIP. **b** The similarities and differences of down-regulated expressed genes in pancreatic cancer gene datasets GSE16515, GSE32676 and GSE71989. **c** JADE-1 was both the target gene of miR-135b and the down-regulated expressed gene of pancreatic cancer. **d**, **e** The heat map of GSE16515 and GSE71989 gene expression datasets for the top 60 down-regulated differential genes (the abscissa represents the sample number, the ordinate represents the differentially expressed genes, the histogram at the upper right represents the color scale, and each rectangle in the map corresponds to a sample expression value). **f** The expression of JADE-1 in pancreatic cancer gene dataset GSE32676

To further investigate the molecular mechanism that miR-135b may participate in PC progression, the target genes of miR-135b were predicted from the DIANA, miRDB, miRWalk, and mirDIP, the results of which demonstrated that 1116, 472, 10,132, and 1159 target genes were obtained respectively. Venn map (Fig. 4a) provided the data indicating 192 intersected genes, and these intersected genes were taken as the target genes of miR-135b for subsequent analyses. In the meantime, the differentially expressed genes were screened out from PC gene datasets i.e., GSE16515, GSE32676, and GSE71989, of which 475, 213, and 646 genes were down-regulated in each dataset respectively. Comparisons of these differentially expressed genes from the 3 datasets revealed there were 15 intersected genes (Fig. 4b). Thereafter, these 15 intersected down-regulated genes in PC tissues of the 3 datasets were used as candidate genes which were further aligned with the target genes of miR-135b (Fig. 4c). Only one intersected gene, namely, JADE-1, was identified, which suggested that the low expression of JADE-1 in PC might be regulated by miR-135b. The gene expression heat maps of GSE16515 and GSE71989 datasets are shown in Fig. 4d, e respectively while the expression changes of JADE-1 in GSE32676 are shown in Fig. 4f, which indicated that JADE-1 was poorly expressed in PC tissues. Nevertheless, a previous study has revealed that JADE-1 is an anti-oncogene with the ability to inhibit the AKT pathway [15], which poses significant importance in PC [23]. Therefore, we speculated that miR-135b might target JADE-1 and regulates the growth of PC tissues.

### miR-135b inhibits the expression of JADE-1

**Fig. 5:**
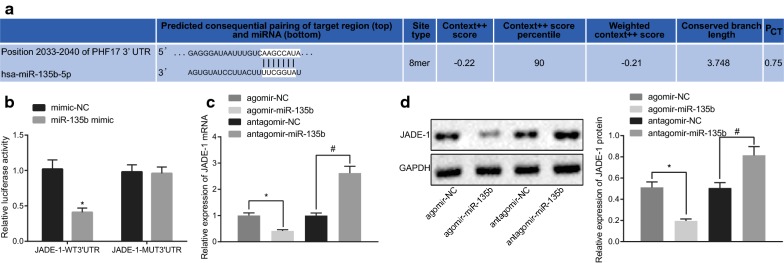
miR-135b suppressed JADE-1 expression specifically. **a** The binding site of miR-135b to JADE-1 (aliasing PHF-17) 3′UTR was analyzed by Targetscan. **b** dual-luciferase reporter gene assay was used to determine the targeting relationship between miR-135b and JADE-1 (*p < 0.05 vs. the agomir-NC group); **c** RT-qPCR was applied to measure JADE-1 mRNA expression in PCSCs of each group. **d** western blot analysis was used to examine the expression of JADE-1 protein in PCSCs of each group. *p < 0.05 vs. The mimic-NC or antagomir-NC group, ^#^*p* < 0.05 vs. the agomir-NC group. The measurement data were expressed as mean ± standard deviation. One-way ANOVA was applied for comparison among multiple groups, followed by Tukey’s post hoc test. The experiment was repeated 3 times

The target gene JADE-1 binding sites of miR-135b were predicted through the bioinformatics online tool Targetscan (Fig. 5a). The dual-luciferase reporter gene assay illustrated that compared to the other 3 groups, the luciferase activity in the JADE-1-WT 3’UTR and miR-135b mimic co-transfected group was significantly decreased (Fig. 5b). RT-qPCR and western blot analysis demonstrated that the JADE-1 expression in the agomir-miR-135b group was significantly lower than that in the agomir-NC group. Moreover, compared to the antagomir-NC group, JADE-1 expression in PCSCs of the antagomir-miR-135b group was markedly increased (*p* < 0.05; Fig. 5c, d). Thus, it was suggested that miR-135b suppressed the expression of JADE-1.

### miR-135b promotes activation of the AKT/mTOR signaling pathway via inhibition of JADE-1

**Fig. 6:**
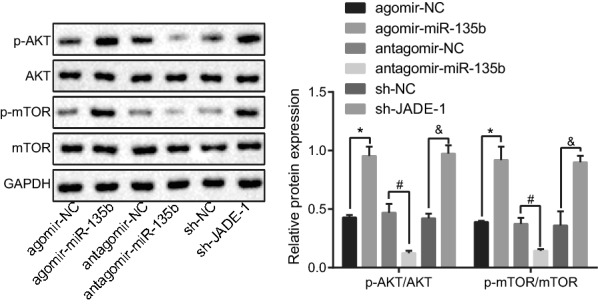
miR-135b promoted activation of AKT/mTOR pathway via inhibition of JADE-1 determined by western blot analysis; *p < 0.05 vs. the agomir-NC group; ^#^*p* < 0.05 vs. the antagomir-NC group; ^&^*p *< 0.05 vs. the sh-NC group; The measurement data were expressed as mean ± standard deviation. One-way ANOVA was applied for comparison among multiple groups, followed by Tukey’s post hoc test. The experiment was repeated 3 times

The AKT/mTOR signaling pathway plays a crucial role in the development of PC [24]. Hence, we set out to investigate the effects conferred by miR-135b and JADE-1 on the AKT/mTOR signaling-pathway related proteins. Western blot analysis documented that overexpressed miR-135b or silencing JADE-1 accelerated the phosphorylation of AKT and mTOR. However, silencing miR-135b led to a drop in the expression of phosphorylated AKT and phosphorylated mTOR, but no significant effect on AKT and mTOR expression was found (Fig. 6, *p* < 0.05). Hence, overexpression of miR-135b activated the AKT/mTOR pathway through inhibition of JADE-1.

### miR-135b activates the AKT/mTOR pathway to promote cell proliferation and restrict apoptosis of PCSCs

**Fig. 7:**
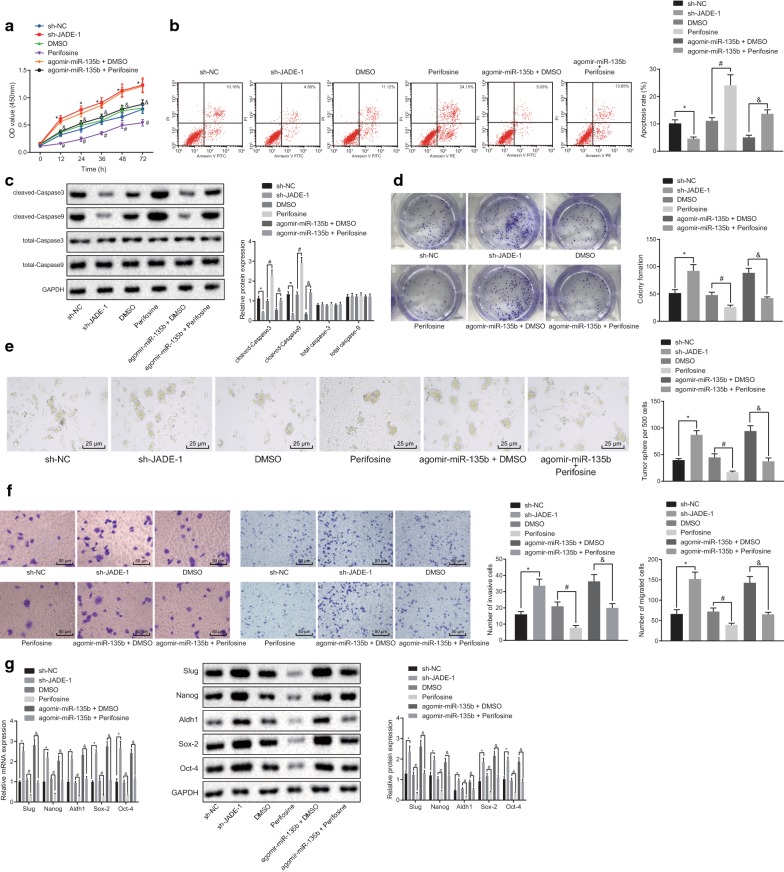
miR-135b activates AKT/mTOR pathway to promote cell proliferation and restrict apoptosis of PCSCs. **a** CCK-8 was used to test the proliferation of PCSCs. **b** Flow cytometry was applied to detect apoptosis. **c** Western blot analysis was utilized to examine the expression of apoptotic protein. **d** Clone forming assay was employed to determine the activity of PCSCs. **e** Onco-spheroids formation assay was used to test the sphere forming ability of PCSCs (×400). **f** Transwell was applied to detect the migration and invasion of PCSCs (×200). **g** Western blot analysis and RT-qPCR were carried out to detect expression of tumor stem cell markers. *p < 0.05 vs. the sh-NC group; ^#^*p* < 0.05 vs. the DMSO group; ^&^*p* < 0.05 vs. the agomir-miR-135b + DMSO group. The measurement data were expressed as the mean ± standard deviation. One-way ANOVA was applied for comparison among multiple groups, followed by Tukey’s post hoc test. Two-way ANOVA was performed to compare the data of cell viability among multiple groups at different time points. Repeated measurement ANOVA was used to compare the data among multiple groups at different time points, followed by the Bonferroni post hoc test. The experiment was repeated 3 times

To investigate whether miR-135b-inhibited JADE-1 promotes the AKT/mTOR pathway to affect the characteristics of PCSCs, CCK-8 assay was first employed to examine the viability of PCSCs. Our results showed that cell viability was enhanced after silencing the JADE-1, but perifosine could reverse the promotion of miR-135b on cell viability (Fig. 7a, *p* < 0.05). Thereafter, flow cytometry was used to examine the apoptosis rate of PCSCs. Our results showed that perifosine enhanced whereas the silencing of JADE-1 significantly reduced the apoptotic rate of the cells. Meanwhile, western blot analysis manifested that the expression of apoptosis-related proteins (cleaved-caspase-3 and cleaved-caspase-9) was decreased accordingly by JADE-1 silencing but enhanced by perifosine, while expression of total caspase-3 and total caspase-9 showed no significant change after different transfections (Fig. 7b, c, *p* < 0.05). Next, the cloning ability of PCSCs was examined, we found that perifosine could reverse the advance effect of miR-135b on PCSC cloning ability. The cloning ability of cells was strengthened after JADE-1 silencing (Fig. 7d, *p* < 0.05). The above results showed that overexpression of miR-135b decreased JADE-1 expression and activated the AKT/mTOR pathway to promote proliferation and inhibit apoptosis of PCSCs.

The stem cell onco-spheroids formation assay described that compared with the sh-NC group, the rate of PCSC sphere-forming in the sh-JADE-1 group was significantly higher. Compared to the DMSO group, the sphere-forming rate of PCSCs in the perifosine group was decreased. Compared with the agomir-miR-135b + DMSO group, the sphere-forming rate in the agomir-miR-135b + perifosine group was significantly decreased (Fig. 7e, *p* < 0.05). Transwell assay was employed to investigate the migration and invasion ability of PCSCs (Fig. 7f, *p* < 0.05). Our results exhibited that compared with the sh-NC group, the migration and invasion abilities of PCSCs in the sh-JADE-1 group were significantly enhanced. Compared with the DMSO group, the migration and invasion of PCSCs in the perifosine group were decreased. Compared with the agomir-miR-135b + DMSO group, the migration and invasion of PCSCs in the agomir-miR-135b + perifosine group were remarkably weakened. Western blot analysis and RT-qPCR were used to detect the mRNA and protein expression of some representative tumor stem cell markers (Sox-2, Oct-4, Nanog, Aldh1, Slug,) and our results showed that the mRNA and protein expression of tumor stem cell markers in the sh-JADE-1 group was significantly higher than that in the sh-NC group. Compared to the DMSO group, the mRNA and protein expression of tumor stem cell markers in the perifosine group was declined. As compared to the agomir-miR-135b + DMSO group, the mRNA and protein expression of tumor stem cell markers in the agomir-miR-135b + perifosine group was significantly declined (Fig. 7g, *p* < 0.05). These results suggested that miR-135b might affect the biological characteristics of PCSCs by targeting JADE-1 to activate the AKT/mTOR pathway.

### miR-135b activates AKT/mTOR pathway to elevate the tumorigenicity of PCSCs in vivo

**Fig. 8:**
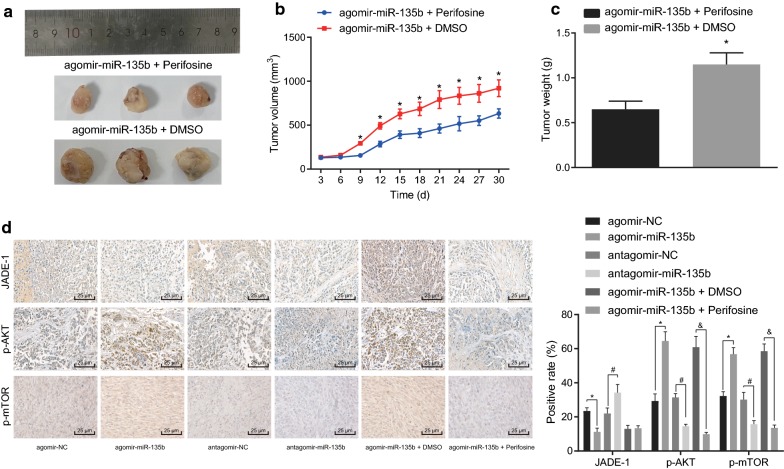
miR-135b activates AKT/mTOR pathway to elevate the tumorigenicity of PCSCs. **a** Macroscopic observation of tumor size. **b** Measurement of tumor volume in nude mice with vernier caliper. **c** Determination of tumor mass in nude mice by weighing method. **d** Immunohistochemistry was performed to detect positive expression of JADE-1, phosphorylated AKT and phosphorylated mTOR (×400); n = 6, *p < 0.05 vs. the agomir-miR-135b + perifosine group; ^#^*p* < 0.05 vs. the antagomir-NC group; ^&^*p* < 0.05 vs. the agomir-miR-135b + DMSO group. n = 6. The measurement data were expressed as mean ± standard deviation. Unpaired student’s *t*-test was used to compare unpaired data between the two groups. Repeated measurement ANOVA was used to compare the data among multiple groups at different time points, followed by the Bonferroni post hoc test

To elucidate the effect of miR-135b activating the AKT/mTOR pathway on the tumorigenicity of PCSCs in vivo, tumor xenografts in nude mice were conducted with PCSCs transfected with agomir-miR-135b + DMSO and the agomir-miR-135b + perifosine. By recording the growth of tumors in subcutaneous tumor-bearing mice, the growth trend of tumors in nude mice was statistically analyzed. The tumor weight was measured by peeling off the tumor mass on the 30th day. Our results indicated that compared with the agomir-miR-135b + perifosine group, PCSCs grew rapidly in the subcutaneous part of nude mice, whereas in the agomir-miR-135b + DMSO group, the tumor volume and weight exhibited significant increase (Fig. 8a–c). Meanwhile, our results of immunohistochemistry documented that in contrast to the agomir-NC group, tumor of mice in the agomir-miR-135b group had reduced JADE-1 positive expression rate and enhanced positive expression rates of phosphorylated AKT and phosphorylated mTOR while the antagomir-miR-135b exhibited the opposite trends as compared to the antagomir-NC (*p* < 0.05). However, JADE-1 positive expression rate did not potently change and positive expression rates of phosphorylated AKT and phosphorylated mTOR were diminished (*p* < 0.05; Fig. 8d). In summary, miR-135b activates the AKT/mTOR pathway, thus enhancing the tumorigenicity of PCSCs in vivo.

## Discussion

PC is considered as the most devastating malignancies known to man, characterized by rarely exhibiting symptoms at the early stage, poor prognosis, rapid growth, and expansion [25]. Hence, a comprehensive understanding of the molecular mechanisms associated with the physiology of PC is urgently required. A recent study has highlighted the critical role of miRNAs in prognostic and therapeutic perspectives for various cancers including PC [26]. The purpose of the present study was to investigate the roles of miR-135b in biological characteristics and tumor growth of PCSCs. Intriguingly, Our data from cancer cell lines and animal experiments demonstrated that miR-135b down-regulated the JADE-1 to promote proliferation and suppress apoptosis of PCSCs via the AKT/mTOR pathway.

Being a member of miRNAs, miR-135b has been reported to be involved in growth in many malignancies. Particularly, a study by Lin et al. has indicated the upregulation of miR-135b in highly invasive non-small-cell lung cancer cells, which ultimately enhances the cancer cell migratory and invasive abilities in vitro [27]. Moreover, Arigoni et al. have denoted the upregulation of miR-135b in basal or normal-like human breast cancers and that it was strongly correlated with patient survival and early metastasis [28]. In gastric cancer cells, miR-135b has also been reported to accelerate cell proliferation and promote other cellular function, including cell invasion, colony formation, migration, and sphere formation in gastric cancer cells [29], which was consistent with the results of our study.

Moreover, here we also attempted to unravel the mechanism by which miR-135b activate the AKT/mTOR pathway to regulate the stem cells by inhibiting JADE-1 expression, which subsequently affects the stemness of PCSCs and promotes tumor growth. Our study identified JADE-1 as a novel gene specifically regulated by miR-135b in PCSCs. As a transcription factor, JADE-1 represents a component of the HBO1 complex characterized by acetylation of histone protein [30]. Interestingly, JADE-1 has been identified as the first member of the JADE protein family containing plant homology domain with the function of suppressing proliferation and advancing apoptosis via the Wnt pathway [31, 32]. The AKT is a well-known protein kinase, encoded by homologs of intracellular retroviruses [33]. Besides, mTOR is widely understood to be an evolutionarily conservative nutrition-sensitive protein kinase capable of adjusting the growth and metabolism of eukaryocytes [34]. With the indispensable role in cell metabolism and other critical functions, the AKT/mTOR pathway has been regarded as an important target for treating malignancies [35]. A previous study has suggested that the AKT pathway could be activated by up-regulating miR-135b, thus suppressing the development of colorectal cancer cells [19]. Additionally, overexpression of miR-135b elevated the expression of phosphorylated PI3K, AKT, and mTOR in hypoxia-treated human umbilical vein endothelial cells, thus activating the PI3K/AKT/mTOR pathway [36], which was concurred with our results. Notably, decreased expression of JADE-1 (a major regulator of the AKT pathway) has been attributed to the poor prognosis and activation of AKT in the renal cancer cell, thus, increasing the cell invasion [15]. Of note, the AKT/mTOR pathway has been indicated to play a critical role in accelerating the proliferation and survival of PC cells [37]. Peculiarly, miR-135b has been illustrated as a promoter of cisplatin resistance in gastric cancer cells by activating the MAPK pathway [38]. Moreover, miR-135b has also been reported to activate the PI3K/AKT pathway to promote chemoresistance of colorectal cancer [39]. Considering these above-described findings we may speculate that miR-135b may possess an oncogenic role in PC to regulate these pathways, but further studies are required to confirm these findings.

Moreover, the present study has also indicated that miR-135b-mediated activation of the AKT/mTOR pathway via down-regulation of JADE-1, further enhancing the expression of the stemness-related genes, such as Sox2, Nanog, and Oct-4. However, scarce studies are determining the relationship between miR-135b and the stemness-linked genes, except for certain miRNAs. For example, miR-200c has been reported to negatively regulate the expression of Sox-2 to suppress the AKT pathway [40]. The expression of miR-148a can suppress the Oct-4/Sox-2-stimulated stem-cell like tumor cell proliferation in glioblastoma based on the suppression of stem cell markers [41]. Oct-4 has been reported as a major transcription factor in regulating the self-renewal and versatility in embryonic stem cells [42].

Importantly, Sox2 belongs to the SRY-related high mobility group box (SOX) gene family that encodes transcription factors and possesses a single HMG DNA-binding field [43]. Furthermore, removal of Sox2 in pancreatic ductal glandular cancer cells enhanced the inhibition activity of small-molecule aiming at AKT [44]. Furthermore, Nanog is viewed as one of the key transcription factors in preserving self-renewal and the undifferentiated condition of pluripotent stem cells [45]. Thus, Sox2, Nanog, and Oct-4 commonly preserve the regulatory system and maintain the versatility and self-renewal of stem cells [46]. Therefore, we concluded that inhibition of JADE-1 on the AKT/mTOR pathway affects the stemness of PCSCs and suppresses tumor growth.

## Conclusion

**Fig. 9:**
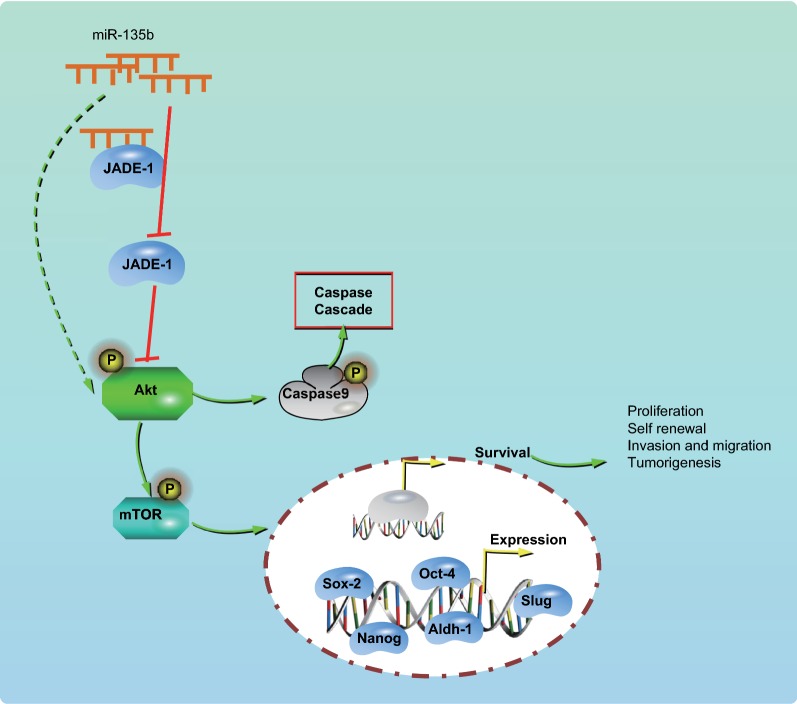
miR-135b-regulation of JADE-1 inhibited the growth of cancer stem cells and tumors in PC via the AKT/mTOR pathway. miR-135b could promote sphere-forming, cloning, invasion, migration, tumor formation of PCSCs in vivo. The mechanism may be performed through inhibiting JADE-1 and activating AKT/mTOR pathway, thus affecting the stemness of PCSCs and promoting tumor growth

Taken together, the critical findings of our study suggest that miR-135b negatively regulated the JADE-1 and controlled the stem cell activity in PC via the Akt/mTOR pathway (Fig. 9). Notably, these findings highlight the potential of miR-135b as a promising therapeutic target for PC. However, due to the lack of comprehensive data about the correlation between miR-135b with JADE-1 and the AKT/mTOR pathway in PC progression, further investigations are indispensable to reveal the underlying mechanism by which miR-135b influences the PCSCs.

## Supplementary information

**Additional file 1: Table S1.** Specific information of pancreatic cancer datasets. **Table S2.** LogFC and p values of top 20 differentiated miRNA expression in pancreatic cancer in GSE41369 dataset.

## Data Availability

The datasets generated/analyzed during the current study are available.

## Abbreviations

miRNAs: MicroRNAs
PC: Pancreatic cancer
PCSCs: Pancreatic cancer stem cells
GEO: Gene Expression Omnibus
DMSO: Dimethyl sulphoxide
WT: Wild-type
MUT: Mutant-type
ATCC: American Type Culture Collection
Aldh1: Acetaldehyde dehydrogenases
TBST: Tris-buffered saline tween
ECL: Electrochemiluminescence
