# Supplementary material for: Silencing of microRNA-135b inhibits invasion, migration, and stemness of CD24+CD44+ pancreatic cancer stem cells through JADE-1-dependent AKT/mTOR pathway
Source: Cancer Cell Int. 2020 Apr 25;20:134. doi: 10.1186/s12935-020-01210-1 (PMC7183669; doi:10.1186/s12935-020-01210-1)
Supplement: Supplementary file 1 — Additional file 1: Table S1. Specific information of pancreatic cancer datasets. Table S2. LogFC and p values of top 20 differentiated miRNA expression in pancreatic cancer in GSE41369 dataset. [file 12935_2020_1210_MOESM1_ESM.docx]

**Additional file 1: Table S1.** Specific information of pancreatic cancer datasets

| Accession | Platform | Organism | miRNA/gene | Sample |
| --- | --- | --- | --- | --- |
| GSE41369 | GPL16142 | Homo sapiens | miRNA | 9 normal pancreatic tissue samples and 9 pancreatic cancer tissue samples |
| GSE16515 | GPL570 | Homo sapiens | Coding gene | 36 pancreatic tumor tissues and 16 normal tissues |
| GSE32676 | GPL570 | Homo sapiens | Coding gene | 7 benign pancreas and 25 pancreatic cancer tissues |
| GSE71989 | GPL570 | Homo sapiens | Coding gene | 13 pancreatic cancer tissues and 8 normal pancreatic tissues |

**Additional file 1: Table S2.** LogFC and p values of top 20 differentiated miRNA expression in pancreatic cancer in GSE41369 dataset

|  | logFC | AveExpr | T | P.Value | adj.P.Val | B |
| --- | --- | --- | --- | --- | --- | --- |
| hsa-miR-135b | 2.820 | 6.126 | 8.360 | 5.97E-08 | 4.38E-05 | 8.423 |
| hsa-miR-221 | 2.180 | 7.354 | 6.506 | 2.45E-06 | 0.000900418 | 4.941 |
| hsa-miR-10a | 2.843 | 7.523 | 6.088 | 6.04E-06 | 0.001350124 | 4.086 |
| hsa-miR-197 | 2.185 | 4.616 | 5.997 | 7.36E-06 | 0.001350124 | 3.898 |
| hsa-miR-145 | 3.240 | 10.028 | 5.874 | 9.64E-06 | 0.001415669 | 3.640 |
| hsa-miR-490-3p | 1.885 | 4.872 | 5.731 | 1.33E-05 | 0.001621111 | 3.337 |
| hsa-miR-21 | 2.992 | 13.598 | 5.492 | 2.26E-05 | 0.002368585 | 2.828 |
| hsa-miR-484 | 1.834 | 6.145 | 5.422 | 2.65E-05 | 0.002429472 | 2.676 |
| hsa-miR-223 | 2.932 | 8.937 | 5.319 | 3.34E-05 | 0.00253171 | 2.453 |
| hsa-miR-630 | -4.216 | 8.470 | -5.305 | 3.45E-05 | 0.00253171 | 2.423 |
| hsa-miR-302c | -1.369 | 4.239 | -5.240 | 4.00E-05 | 0.002665867 | 2.283 |
| hsa-miR-342-3p | 2.140 | 8.294 | 5.196 | 4.42E-05 | 0.002706011 | 2.185 |
| hsa-miR-125a-5p | 2.485 | 7.018 | 5.123 | 5.22E-05 | 0.002949421 | 2.026 |
| hsa-miR-1975 | 2.294 | 5.772 | 4.959 | 7.61E-05 | 0.003847923 | 1.667 |
| hsa-miR-27a | 3.640 | 7.435 | 4.932 | 8.10E-05 | 0.003847923 | 1.607 |
| hsa-miR-331-3p | 1.834 | 5.867 | 4.916 | 8.39E-05 | 0.003847923 | 1.573 |
| hsa-miR-140-3p | 1.498 | 4.663 | 4.866 | 9.41E-05 | 0.004063318 | 1.463 |
| hsa-miR-337-3p | 1.308 | 5.438 | 4.800 | 0.000109766 | 0.004437182 | 1.316 |
| hsa-miR-142-5p | 2.625 | 7.783 | 4.780 | 0.000114859 | 0.004437182 | 1.273 |
| hsa-miR-190b | 1.364 | 4.476 | 4.587 | 0.000179498 | 0.00658758 | 0.846 |

Note: miRNA, microRNA
